# Psychometric Properties of the Persian Version of the Mind Excessively Wandering Scale in Iranian Adolescents: A Validation Study

**DOI:** 10.1002/brb3.71824

**Published:** 2026-10-06

**Authors:** Mina Hasanpour, Kousar Dadkhah Tehrani, Amir Sam Kianimoghadam, Yalda Daneshi Hir, Abolfazl Chekaneh Bajestani, Shima Hasanpour, Maryam Maadi Esfahan

**Affiliations:** ^1^ Department of Clinical Psychology School of Medicine Shahid Beheshti University of Medical Sciences Tehran Iran; ^2^ Department of Psychology Birjand Branch Islamic Azad University Birjand Iran; ^3^ Department of Electrical & Computer Engineering Texas A&M University College Station Texas USA

**Keywords:** adolescent, Iran, mind wandering, psychometric, questionnaires, validation study

## Abstract

**Purpose:**

The Mind Excessively Wandering Scale (MEWS) assesses excessive mind‐wandering. This study evaluated the psychometric properties of the Persian 12‐item MEWS (MEWS‐12) among Iranian adolescents, focusing on factor structure, internal consistency, associations with external measures, known‐groups validity, and gender measurement invariance.

**Method:**

In this cross‐sectional study, 506 adolescents aged 12–18 years completed the MEWS and measures of rumination, attentional control, mindfulness, depressive and anxiety symptoms, and positive and negative affect. All 15 original MEWS items were administered, with the developer‐recommended 12‐item version designated for primary analyses. The sample was randomly divided, with stratification by gender, into independent exploratory factor analysis (EFA; *n* = 254) and confirmatory factor analysis (CFA; *n* = 252) subsamples.

**Finding:**

Parallel analysis favored a two‐factor EFA solution accounting for 54% of the variance, with a factor correlation of 0.73. In CFA, the correlated two‐factor model showed better fit than the unidimensional model, *χ* ^2^(53) = 122.89, CFI = 0.971, TLI = 0.964, RMSEA = 0.072, 90% CI [0.056, 0.089], and SRMR = 0.054, although the factors were highly correlated (*r* = 0.858). Internal consistency was good (*α* = 0.893; *ω* = 0.917). Higher MEWS‐12 scores were associated with greater depressive and anxiety symptoms, rumination, and negative affect, and with lower mindfulness and positive affect; the association with self‐reported attentional control was small and non‐significant. Adolescents at or above the selected GAD‐7 and PHQ‐9 symptom thresholds had higher MEWS‐12 scores than those below the respective thresholds. Gender invariance analyses provided preliminary support for comparable measurement properties across girls and boys.

**Conclusion:**

The findings provide preliminary evidence regarding the psychometric properties of the Persian MEWS‐12 among Iranian adolescents. The two‐factor representation should be interpreted cautiously given the substantial factor overlap. Further replication in independent samples and evaluation in longitudinal and clinically characterized studies are warranted.

## Graphical abstract

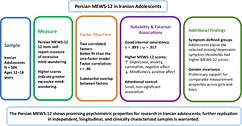

## 1 Introduction

Mind‐wandering refers to a shift of attention away from ongoing tasks toward internally generated thoughts (Smallwood and Schooler 2015; Vannucci et al. 2020). A key distinction has been made between deliberate and spontaneous mind‐wandering, with the latter involving unintentional intrusions of task‐unrelated thoughts (Seli et al. 2016; Vannucci et al. 2020). Spontaneous mind‐wandering may be particularly relevant to excessive forms of mind‐wandering, especially when such thoughts are frequent, intrusive, and difficult to control (Mowlem et al. 2019). Although mind‐wandering may support adaptive functions such as future planning and problem‐solving, frequent or poorly controlled mind‐wandering has been associated with poorer sustained attention, reduced task performance, and emotional symptoms (Luo et al. 2016; Martinon et al. 2019; Mooneyham and Schooler 2013; Smallwood and Schooler 2015). These findings highlight the relevance of reliable assessment of excessive mind‐wandering during adolescence.

Adolescence is a developmental period in which attentional control, emotion regulation, metacognition, and self‐reflective processes are still maturing (Sawyer et al. 2018; Webb et al. 2022). Excessive mind‐wandering has also been associated with depressive and anxiety symptoms, as well as attention‐deficit/hyperactivity disorder (ADHD)‐related symptoms (Fell et al. 2023; Lanier et al. 2021; Welhaf et al. 2024). Together, these developmental and clinical considerations may influence how excessive mind‐wandering is experienced and reported during adolescence.

Much of the available research on mind‐wandering and its assessment has focused on adult populations, whereas adolescence has received comparatively less attention (Webb et al. 2021). Given the developmental changes in attentional control, metacognition, and emotion regulation during adolescence, evidence derived from adult samples cannot be assumed to generalize directly to adolescent populations. Accordingly, psychometric evidence obtained in adolescent samples is needed before extending conclusions derived primarily from adult studies to this population.

Several instruments assess mind‐wandering, including the Mind Wandering Questionnaire (MWQ), which measures the general frequency of task‐unrelated thoughts, and the Mind Wandering Spontaneous (MW‐S) and Mind Wandering Deliberate (MW‐D) scales, which distinguish unintentional from intentional mind‐wandering (Carriere et al. 2013; Seli et al. 2016). In contrast, the Mind Excessively Wandering Scale (MEWS) specifically assesses excessive mind‐wandering, emphasizing frequent, intrusive, and difficult‐to‐control internally generated thoughts (Mowlem et al. 2019). The MEWS was initially developed as a 15‐item measure and was subsequently reduced to a 12‐item version recommended for future research (Mowlem et al. 2019). The recommended 12‐item MEWS (MEWS‐12) was evaluated as a unidimensional measure in the original validation, whereas subsequent work also evaluated a correlated three‐factor representation of the same 12 items (Morillas‐Romero et al. 2023; Mowlem et al. 2019). Although previous studies have examined the psychometric properties of the MEWS in adult, cross‐cultural, and ADHD‐related samples, evidence regarding its psychometric performance in Iranian adolescents remains limited (Dekkers et al. 2025; Morillas‐Romero et al. 2023; Mowlem et al. 2019).

Cultural and linguistic contexts may affect the interpretation of translated self‐report items and the extent to which psychometric properties are retained across settings. Therefore, translated or adapted measures cannot be assumed to retain their psychometric properties without systematic evaluation (Beaton et al. 2000; Milfont and Fischer 2010). To address this gaps, the present study aimed to evaluate the psychometric properties of the Persian MEWS‐12 among Iranian adolescents, including its factor structure, internal consistency, associations with external measures, and known‐groups validity; measurement invariance across gender was examined as a secondary analysis.

## 2 Methods

### 2.1 Participants

This cross‐sectional psychometric study included 506 adolescents aged 12–18 years. Of 562 initial respondents, 56 were excluded because of incomplete surveys or invalid response patterns, resulting in a final analytic sample of 506 participants. Adolescents were recruited between fall 2024 and winter 2025 through convenience sampling from local schools in Iran and social media platforms targeting Persian‐speaking adolescents. Eligibility criteria included age between 12 and 18 years, Persian fluency, and informed consent/assent.

### 2.2 Sample Size Justification

The target sample size was determined before data collection based on the 15‐item MEWS originally administered. Published recommendations for psychometric and factor‐analytic studies vary substantially, with commonly cited participant‐to‐item ratios ranging from approximately 3:1 to 20:1 (Anthoine et al. 2014; Mundfrom et al. 2005). We aimed to recruit approximately 500 adolescents to obtain a large sample for psychometric evaluation while allowing for exclusions after data screening. Following data screening, 506 participants were retained. For evaluation of the factor structure, the final sample was randomly divided, with stratification by gender, into independent EFA (*n* = 254) and CFA (*n* = 252) subsamples. Each subsample included approximately 17 participants per originally administered item and 21 participants per item for the primary MEWS‐12 analyses.

### 2.3 Procedure

Data were collected using online and paper‐based questionnaires. Participants completed the Persian MEWS along with measures of rumination, attentional control, mindfulness, depression, anxiety, and affect. Online responses were collected through secure survey links, whereas paper‐based questionnaires were administered in school settings. Anonymity and confidentiality were maintained throughout data collection.

### 2.4 Data Screening and Missing Data

Data were screened for missing values, out‐of‐range responses, and invalid response patterns prior to analysis. Invalid responses included invariant responding across extended item blocks, logically inconsistent responses, and implausibly short completion patterns. After exclusions, no missing data were observed for the MEWS or other psychometric scale items in the final analytic sample. Missing demographic data were limited to selected background variables, particularly family income, parental education, and birth order, which some participants may have perceived as sensitive despite the anonymous and confidential nature of data collection; therefore, psychometric analyses were conducted on all 506 participants, while demographic summaries were based on available data.

### 2.5 Translation and Cultural Adaptation of the Persian MEWS

The Persian version was developed using a forward–backward translation procedure based on established principles of translation and cross‐cultural adaptation (Brislin 1970; Mokkink et al. 2010). Two bilingual psychologists independently translated the original English version into Persian, and discrepancies were resolved through consensus. The reconciled version was back‐translated into English by two independent bilingual translators blinded to the original instrument. Semantic and conceptual equivalence between the original and back‐translated versions was evaluated by the translation team and a Persian‐speaking psychologist. The pre‐final version was reviewed by a panel of psychologists and psychometricians to assess clarity and cultural relevance. The experts qualitatively reviewed each item for clarity, conceptual consistency with excessive mind‐wandering, and cultural appropriateness for Persian‐speaking adolescents. The pre‐final version was pilot‐tested with 25 Persian‐speaking adolescents to assess item comprehensibility.

### 2.6 Measures

#### 2.6.1 The Mind Excessively Wandering Scale

The MEWS was developed by Mowlem et al. (2019) to assess excessive mind‐wandering in individuals with and without ADHD. The original MEWS comprised 15 items rated on a 4‐point Likert scale from 0 (“never or rarely”) to 3 (“almost always or constantly”), with higher scores indicating greater excessive mind‐wandering. In the present study, all 15 original MEWS items were administered. However, following the scale developers’ recommendation, the primary psychometric analyses focused on the 12‐item version of the MEWS. In their original validation study, Mowlem et al. (2019) recommended excluding Items 6, 10, and 14 from scoring in future research because these items showed comparatively weaker psychometric performance and reflected either a functional consequence of excessive mental activity (sleep difficulty; Item 6) or strategies used to manage thoughts (distraction and substance use; Items 10 and 14), rather than the core phenomenology of excessive mind‐wandering. Their removal did not meaningfully reduce the scale's discriminative ability. Accordingly, analyses of the 12‐item version were designated as primary in the present study, whereas analyses including all 15 items were conducted and reported as exploratory in the Supporting Information. The original validation study supported a unidimensional structure and reported good internal consistency (Cronbach's *α* > 0.78) and satisfactory test–retest reliability (*r* = 0.63) (Mowlem et al. 2019).

#### 2.6.2 The Ruminative Responses Scale

The Ruminative Responses Scale (RRS), developed by Nolen‐Hoeksema and Morrow (1991), is a 22‐item measure designed to assess individuals’ tendency to engage in ruminative thinking in response to depressed mood. Items are rated on a 4‐point Likert scale ranging from 1 (“almost never”) to 4 (“almost always”), with higher scores indicating greater rumination. The original validation study reported excellent internal consistency for the scale (Cronbach's *α* > 0.88. Adolescent‐specific psychometric evidence for the Persian RRS in Iran is limited; however, studies in non‐Western adolescent populations have reported satisfactory reliability (Cronbach's *α* = 0.92–0.95) (Liang and Lee 2019; Shin et al. 2015). In addition, studies conducted in Iranian samples aged 16 years and older have reported acceptable internal consistency coefficients across subscales (Cronbach's *α* = 0.88–0.92) (Mansouri et al. 2012). In the present sample, the RRS exhibited excellent internal consistency (Cronbach's *α* = 0.95).

#### 2.6.3 The Attentional Control Scale

The Attentional Control Scale (ACS), developed by Derryberry and Reed (2002), is a 20‐item measure designed to assess individual differences in attentional control, particularly attentional focusing and attentional shifting. Items are rated on a 4‐point Likert scale ranging from 1 (“almost never”) to 4 (“always”), with higher scores indicating greater attentional control. Previous studies supported the reliability and construct validity of the ACS, with good internal consistency for the original version (Cronbach's *α* = 0.84) (Ólafsson et al. 2011). The Persian ACS has shown a two‐factor structure, acceptable internal consistency (0.66–0.78), and test–retest reliability (0.76–0.82) in Iranian samples (Abasi et al. 2017). In the present sample, the ACS showed modest internal consistency (Cronbach's *α* = 0.63).

#### 2.6.4 The Five Facet Mindfulness Questionnaire

The Five Facet Mindfulness Questionnaire (FFMQ), developed by Baer et al. (2006), is a 39‐item measure designed to assess five dimensions of mindfulness. Items are rated on a 5‐point Likert scale ranging from 1 (“never or very rarely true”) to 5 (“very often or always true”), with higher scores indicating greater mindfulness. The original study reported internal consistency coefficients ranging from 0.75 to 0.91 across subscales. Previous Iranian studies using the Persian FFMQ have reported internal consistency coefficients ranging from 0.55 to 0.80 and test–retest coefficients ranging from 0.57 to 0.80 (Heydarinasab 2013; Khanjani et al. 2022). In the present sample, the FFMQ demonstrated acceptable internal consistency (Cronbach's *α* = 0.71).

#### 2.6.5 The Patient Health Questionnaire (PHQ‐9)

The Patient Health Questionnaire‐9 (PHQ‐9), developed by Kroenke et al. (2001), is a 9‐item measure designed to assess depressive symptoms over the previous 2 weeks. Items are rated on a 4‐point Likert scale ranging from 0 (“not at all”) to 3 (“nearly every day”), with higher scores indicating greater depressive symptom severity. The original version showed excellent internal consistency (Cronbach's *α* = 0.89). Although the Persian PHQ‐9 has not been specifically standardized for Iranian adolescents, the adult Persian version has shown acceptable reliability in Iranian samples (Cronbach's *α* = 0.86) (Farrahi et al. 2021). For the symptom‐based group comparisons, a score of ≥ 10 was used to define the higher‐symptom group, consistent with previous PHQ‐9 studies (Kroenke et al. 2001; Mohamadian et al. 2022). In the present sample, the PHQ‐9 showed excellent internal consistency (Cronbach's *α* = 0.89).

#### 2.6.6 The Generalized Anxiety Disorder (GAD‐7)

The Generalized Anxiety Disorder Scale‐7 (GAD‐7), developed by Spitzer et al. (2006), is a 7‐item measure designed to assess generalized anxiety symptoms over the previous 2 weeks. Items are rated on a 4‐point Likert scale ranging from 0 (“not at all”) to 3 (“nearly every day”), with higher scores indicating greater anxiety severity. The original version showed excellent internal consistency (Cronbach's *α* = 0.92) and satisfactory test–retest reliability (*r* = 0.83). Although the Persian GAD‐7 has not been specifically standardized for Iranian adolescents, the adult Persian version has shown acceptable reliability in Iranian samples (Cronbach's *α* = 0.85; test–retest reliability = 0.48) (Naeinian et al. 2011). For the symptom‐based group comparisons, a score of ≥ 10 was used to define the higher‐symptom group, consistent with previous GAD‐7 studies (Rashtbari et al. 2019; Spitzer et al. 2006). In the present sample, the GAD‐7 showed good internal consistency (Cronbach's *α* = 0.87).

#### 2.6.7 The Positive and Negative Affect Schedule (PANAS)

The Positive and Negative Affect Schedule (PANAS), developed by Watson et al. (1988), is a 20‐item measure designed to assess positive and negative affect. Each subscale includes 10 items rated on a 5‐point Likert scale ranging from 1 (“very slightly or not at all”) to 5 (“extremely”), with higher scores indicating greater intensity of the corresponding affect. The original version showed strong internal consistency for both positive affect (Cronbach's *α* = 0.86–0.90) and negative affect (Cronbach's *α* = 0.84–0.87) (Watson et al. 1988). The Persian version used in the present study has shown acceptable reliability in Iranian samples, with Cronbach's alpha coefficients of 0.83 for positive affect and 0.78 for negative affect (Shokri et al. 2014). In the present sample, the PANAS demonstrated acceptable internal consistency (Cronbach's *α* = 0.79).

#### 2.6.8 Use of Persian External Measures in the Adolescent Sample

The available Persian‐language versions of the FFMQ, RRS, ACS, PHQ‐9, GAD‐7, and PANAS were used as external measures in the present study. Relevant psychometric evidence from Iranian samples is summarized in the corresponding instrument descriptions. Internal consistency was re‐estimated for each measure in the present adolescent sample.

### 2.7 Statistical Analyses

All analyses were conducted in R version 4.5.1 (R Core Team 2025). Psychometric analyses focused on the MEWS‐12; exploratory analyses of all 15 administered items are reported in the Supporting Information.

Descriptive statistics were computed for MEWS‐12 items and total scores. Internal consistency was assessed using Cronbach's alpha, McDonald's omega, corrected item–total correlations, and alpha‐if‐item‐deleted values. Pearson correlations with external measures were used to examine construct validity, with expected positive associations with depressive and anxiety symptoms, rumination, and negative affect, and expected negative associations with mindfulness, positive affect, and attentional control.

The gender‐stratified sample was randomly split into independent EFA (*n* = 254) and CFA (*n* = 252) subsamples. EFA used polychoric correlations, minimum residual extraction, and oblique rotation for the ordinal MEWS items. Factorability was assessed using the Kaiser–Meyer–Olkin measure and Bartlett's test; factor retention was guided by parallel analysis, eigenvalues, interpretability, and loading patterns. The Bayesian information criterion (BIC) was also considered for comparing exploratory factor solutions, with l ower values indicating a more parsimonious fit.

CFA in the independent confirmatory subsample was conducted using the lavaan package (Rosseel et al. 2026) with the weighted least squares mean‐ and variance‐adjusted estimator (WLSMV) for ordinal indicators. Based on prior MEWS research and the present EFA, three CFA models were evaluated: the original unidimensional 12‐item model (Mowlem et al. 2019), the correlated three‐factor 12‐item model examined by Morillas‐Romero et al. (2023), and the correlated two‐factor model derived from the present EFA. A bifactor model was also examined as an additional model comparison. The unidimensional and correlated two‐factor CFA models were compared using a robust scaled chi‐square difference test appropriate for ordinal data estimated with WLSMV. Fit was evaluated using chi‐square statistic (*χ* ^2^), the Comparative Fit Index (CFI), Tucker–Lewis index (TLI), root mean square error of approximation (RMSEA; 90% CI), and standardized root mean square residual (SRMR). CFI/TLI ≥ 0.90 and RMSEA/SRMR ≤ 0.08 indicated acceptable fit; indices were interpreted jointly and across theoretically plausible models rather than as rigid cutoffs (Hu and Bentler 1999; Kline 2016).

As a secondary analysis, multigroup CFA tested gender invariance across configural, equal‐threshold, and equal‐loading‐and‐threshold models. Invariance was considered supported when imposing additional constraints did not reduce CFI by more than 0.010 or increase RMSEA by more than 0.015 (Chen 2007).

Known‐groups validity was examined by comparing MEWS‐12 scores between adolescents scoring below versus at or above a score of 10 on the GAD‐7 and PHQ‐9. Welch's *t*‐tests, Hedges’ *g* (95% CIs), and sensitivity Wilcoxon rank‐sum tests were used. These comparisons were intended to examine whether MEWS‐12 scores differentiated adolescents with relatively higher versus lower anxiety and depressive symptoms; they were not used to establish diagnostic or screening cutoffs for the MEWS‐12.

## 3 Results

Of 562 initial respondents, 56 were excluded because of incomplete surveys or patterned/invalid responses, leaving a final analytic sample of 506 adolescents. No missing responses were observed for the psychometric scale items; missing data were limited to selected demographic variables and are reported in Table 1.

**TABLE 1:** Demographic characteristics of adolescent participants (*N* = 506).

Variable	*n*/mean	%/SD
**Age, years**	15.21	1.48
**Gender**		
Female	265	52.37
Male	241	47.63
**Birth order**		
Only child	30	5.93
First	222	43.87
Second	122	24.11
Third	15	2.96
Fourth	6	1.19
Missing/not reported	111	21.94
**Family income, million IRR/month**		
10–20	229	45.26
20–30	104	20.55
30–40	33	6.52
40–50	25	4.94
≥ 50	4	0.79
Missing/not reported	111	21.94
**Parental education**		
**Education level**	**Father *n* (%)**	**Mother *n* (%)**
None	31 (6.13)	40 (7.91)
Middle school	37 (7.31)	47 (9.29)
High school	176 (34.78)	184 (36.36)
Bachelor's	117 (23.12)	108 (21.34)
Master's	32 (6.32)	14 (2.77)
Ph.D.	2 (0.40)	2 (0.40)
Missing/not reported	111 (21.94)	111 (21.94)

*Note*: Percentages are based on the total analytic sample (*N* = 506). Missing or not reported values are presented where applicable.

### 3.1 Descriptive Statistics

Descriptive analyses were performed for the 12 Persian MEWS items. Item means ranged from 0.94 to 1.92, with standard deviations between 0.97 and 1.11. Skewness ranged from −0.43 to 0.74, and kurtosis ranged from −1.32 to −0.65.

### 3.2 Sample Splitting and Factorability of the Data

To reduce the risk of capitalizing on chance, the full sample (*N* = 506) was randomly divided into independent exploratory (EFA; *n* = 254) and confirmatory (CFA; *n* = 252) subsamples. A gender‐stratified random‐splitting procedure was used to preserve the gender distribution across subsamples. The EFA subsample included 133 girls and 121 boys, whereas the CFA subsample included 132 girls and 120 boys. The two subsamples also showed comparable age characteristics (EFA: M = 15.16, SD = 1.49; CFA: M = 15.27, SD = 1.47).

Each subsample included approximately 21 participants per item for the MEWS‐12. Although sample size adequacy for factor analysis cannot be determined solely by a fixed participant‐to‐item ratio, it is influenced by features such as factor loadings, communalities, and the number of indicators per factor (MacCallum et al. 1999; Mundfrom et al. 2005). Empirical evaluation of the EFA subsample supported its suitability for factor analysis: the overall Kaiser–Meyer–Olkin index was 0.92, with item‐level measures of sampling adequacy ranging from 0.88 to 0.95. In addition, Bartlett's test of sphericity was significant, *χ* ^2^(66) = 1634.04, *p* < 0.001, indicating that the polychoric correlation matrix was factorable.

### 3.3 Exploratory Factor Analysis

Exploratory factor analysis (EFA) was conducted in the exploratory subsample (*n* = 254) using a polychoric correlation matrix, minimum residual (minres) extraction, and oblimin rotation. Because the latent dimensions of excessive mind‐wandering were expected to be related, oblimin rotation was applied. Parallel analysis based on the polychoric correlation matrix suggested a two‐factor solution. Accordingly, one‐, two‐, and three‐factor solutions were examined. The two‐factor solution was retained based on the parallel‐analysis findings, its improved fit relative to the one‐factor solution, its lower BIC than the one‐ and three‐factor solutions, the interpretability of its loading pattern, and parsimony.

Although all items showed moderate‐to‐high loadings in the one‐factor solution (0.63–0.78), its fit was comparatively limited (TLI = 0.891, RMSEA = 0.101, 90% CI [0.086, 0.117], BIC = −105.77). The two‐factor solution accounted for 54% of the variance and showed improved fit relative to the one‐factor solution (TLI = 0.931, RMSEA = 0.080, 90% CI [0.062, 0.098], BIC = −125.12). The first factor was primarily defined by Items 1–5, 7, and 13, whereas the second factor was primarily defined by Items 8, 9, 11, 12, and 15. Pattern coefficients ranged from 0.51 to 0.88 for the first factor and from 0.46 to 0.91 for the second factor. The two factors were strongly correlated at *r* = 0.73 (Table 2).

**TABLE 2:** Pattern coefficients and communalities for the two‐factor exploratory solution of the Persian MEWS‐12.

Item	Factor 1	Factor 2	*h* ^2^
Item 1	0.51	0.30	0.58
Item 2	0.54	—	0.50
Item 3	0.64	—	0.44
Item 4	0.64	—	0.63
Item 5	0.88	—	0.61
Item 7	0.67	—	0.49
Item 8	—	0.58	0.52
Item 9	0.38	0.46	0.61
Item 11	—	0.91	0.78
Item 12	—	0.49	0.43
Item 13	0.51	—	0.43
Item 15	—	0.72	0.52

*Note*: Exploratory factor analysis was conducted using minimum residual extraction on a polychoric correlation matrix (*n* = 254), with oblimin rotation. Pattern coefficients smaller than 0.30 are omitted. The factors correlated at *r* = 0.73. Factor 1 explained 30% and Factor 2 explained 24% of the variance (54% cumulatively).

A three‐factor solution explained 57% of the variance and showed somewhat better TLI and RMSEA values (TLI = 0.941, RMSEA = 0.074, 90% CI [0.053, 0.095]); however, parallel analysis did not support a three‐factor solution, its BIC was less favorable (−103.87), and cross‐loadings were observed, particularly for Items 8 and 9. Accordingly, the two‐factor solution was carried forward for evaluation in the independent CFA subsample.

### 3.4 Confirmatory Factor Analysis

Confirmatory factor analysis (CFA) was conducted in the independent confirmatory subsample (*n* = 252) using WLSMV estimation for ordered categorical item responses. The analyses compared the original unidimensional MEWS‐12 model (Mowlem et al. 2019), the correlated two‐factor model derived from the present EFA, and the correlated three‐factor model previously examined for the MEWS‐12 (Morillas‐Romero et al. 2023). Given the substantial correlation expected between the EFA‐derived dimensions, a bifactor model comprising a general excessive mind‐wandering factor and two EFA‐derived specific factors was also examined as an alternative representation. The two‐factor model specified Items 1–5, 7, and 13 on Factor 1 and Items 8, 9, 11, 12, and 15 on Factor 2; the latent factors were allowed to correlate (Figure 1).

**FIGURE 1:**
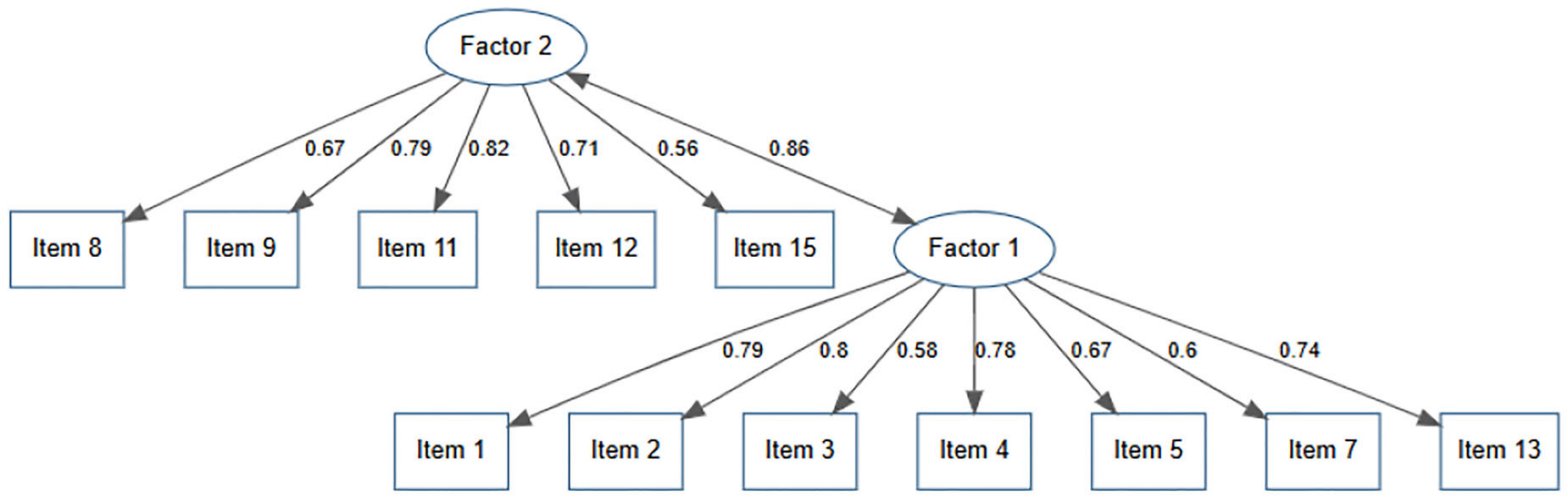
Standardized factor loadings for the two‐factor CFA model of the Persian MEWS‐12. The correlation between the latent factors was 0.86.

As shown in Table 3, the one‐factor model showed comparatively weaker fit. The two‐factor model showed improved fit relative to the one‐factor model, *χ* ^2^(53) = 122.89, CFI = 0.971, TLI = 0.964, RMSEA = 0.072, 90% CI [0.056, 0.089], and SRMR = 0.054. The chi‐square difference test comparing the one‐ and two‐factor models was statistically significant, Δ*χ* ^2^(1) = 27.42, *p* < 0.001.

**TABLE 3:** Fit indices for competing CFA models of the Persian MEWS‐12.

Model	*χ* ^2^	df	CFI	TLI	RMSEA	90% CI for RMSEA	SRMR
One‐factor	163.17	54	0.954	0.944	0.090	[0.074, 0.106]	0.062
Two‐factor	122.89	53	0.971	0.964	0.072	[0.056, 0.089]	0.054
Three‐factor	127.41	51	0.968	0.959	0.077	[0.061, 0.094]	0.053
Bifactor	Did not converge (improper solution)	—	—	—	—	—	—

*Note*: CFA was conducted using WLSMV estimation for ordered categorical item responses in the confirmatory subsample (*n* = 252). The reported statistics for the one‐, two‐, and three‐factor models are robust/scaled fit statistics. The bifactor model did not converge and yielded an improper solution including a negative variance estimate for one specific factor; accordingly, its parameter estimates and fit indices were not interpreted. The robust/scaled chi‐square difference test comparing the one‐ and two‐factor models yielded Δ*χ* ^2^(1) = 27.42, *p* < 0.001.

The three‐factor model did not show a consistent improvement in fit over the two‐factor model, *χ* ^2^(51) = 127.41, CFI = 0.968, TLI = 0.959, RMSEA = 0.077, 90% CI [0.061, 0.094], SRMR = 0.053, and involved a less parsimonious specification.

The correlation between the two factors was *r* = 0.858. In the three‐factor model, two factor correlations were also high (*r*s = 0.889 and 0.894). Considering the parallel‐analysis result, comparative model fit, and parsimony, the correlated two‐factor model was retained as the structural representation carried forward in the present study.

A bifactor model comprising a general excessive mind‐wandering factor and the two EFA‐derived specific factors was additionally tested. The model did not converge and yielded an improper solution, including a negative variance estimate for one specific factor. Accordingly, its parameter estimates and fit indices were not interpreted.

The MEWS‐12 constituted the primary scored version evaluated in the present study. Exploratory analyses of the originally administered 15‐item pool are reported in Tables S1 and S2. Parallel analysis suggested a two‐factor solution; however, the resulting structure was not replicated in the independent CFA subsample. Subsequent psychometric analyses therefore focused on the MEWS‐12.

### 3.5 Internal Consistency

Internal consistency of the MEWS‐12 was evaluated in the full sample (*N* = 506). Cronbach's alpha was 0.893, and McDonald's omega, estimated from the polychoric correlation matrix, was 0.917. Corrected item–total correlations ranged from 0.495 to 0.680. Alpha‐if‐item‐deleted ranged from 0.880 to 0.890, with no value exceeding the full‐scale alpha. MEWS‐12 total scores ranged from 0 to 36 (M = 15.65, SD = 8.45).

### 3.6 Associations With External Measures

MEWS‐12 total scores were positively associated with depressive symptoms (PHQ‐9), anxiety symptoms (GAD‐7), rumination (RRS), and negative affect (PANAS‐NA), and were negatively associated with mindfulness (FFMQ) and positive affect (PANAS‐PA). The association between MEWS‐12 scores and attentional control (ACS) was not statistically significant (Table 4).

**TABLE 4:** Correlations with external measures.

External measure	Pearson's *r*	95% CI	*p*
PHQ‐9	0.670	[0.619, 0.715]	< 0.001
GAD‐7	0.686	[0.637, 0.730]	< 0.001
RRS	0.660	[0.608, 0.707]	< 0.001
FFMQ	−0.504	[−0.566, −0.436]	< 0.001
PANAS–positive affect	−0.200	[−0.282, −0.115]	< 0.001
PANAS–negative affect	0.551	[0.487, 0.609]	< 0.001
ACS	0.056	[−0.031, 0.142]	0.206

*Note: N* = 506. Two‐tailed Pearson correlations.

Abbreviations: ACS = Attentional Control Scale; FFMQ = Five Facet Mindfulness Questionnaire; GAD‐7 = Generalized Anxiety Disorder‐7;

PANAS = Positive and Negative Affect Schedule; PHQ‐9 = Patient Health Questionnaire‐9; RRS = Ruminative Responses Scale.

### 3.7 Known‐Groups Validity

Adolescents scoring at or above the selected GAD‐7 and PHQ‐9 symptom thresholds (≥ 10) had higher MEWS‐12 scores than those scoring below the respective thresholds (Table 5). Wilcoxon rank‐sum tests conducted as sensitivity analyses showed the same pattern of significantly higher MEWS‐12 scores among adolescents scoring at or above the selected symptom thresholds for anxiety symptoms (*W* = 12,179.5, *p* < 0.001) and depressive symptoms (*W* = 12,572.5, *p* < 0.001).

**TABLE 5:** Known‐groups comparisons based on GAD‐7 and PHQ‐9 symptom thresholds.

Outcome	Group means (MEWS‐12)	Welch's *t* (df)	*p*	Hedges’ *g* [95% CI]
GAD‐7 ≥ 10	GAD‐7 < 10: 10.96; GAD‐7 ≥ 10: 19.98	−14.2 (501)	< 0.001	1.26 [1.08, 1.45]
PHQ‐9 ≥ 10	PHQ‐9 < 10: 10.61; PHQ‐9 ≥ 10: 19.37	−13.7 (490)	< 0.001	1.20 [1.01, 1.40]

*Note*: Positive Hedges’ *g* values indicate higher MEWS‐12 scores among participants scoring at or above the selected symptom thresholds.

Abbreviation: CI = confidence interval.

### 3.8 Measurement Invariance Across Gender

As a secondary analysis, measurement invariance across gender was examined using multigroup CFA. A sequence of models was tested: a configural model, followed by an equal‐thresholds model, and then an equal‐loadings‐and‐thresholds model. As shown in Table 6, imposing equal thresholds produced negligible changes in fit relative to the configural model (ΔCFI = −0.002, ΔRMSEA = −0.001). Constraining both factor loadings and thresholds to equality also did not meaningfully worsen model fit relative to the equal‐thresholds model (ΔCFI = +0.002, ΔRMSEA = −0.006). These changes met the prespecified criteria for measurement invariance, providing preliminary support for comparability of MEWS‐12 scores across girls and boys.

**TABLE 6:** Measurement invariance of the MEWS‐12 across gender.

Model	*χ* ^2^ (df)	CFI	TLI	RMSEA [90% CI]	SRMR	ΔCFI	ΔRMSEA
Configural	207.31 (102)	0.977	0.970	0.064 [0.051, 0.076]	0.049	—	—
Equal thresholds	228.61 (114)	0.975	0.971	0.063 [0.051, 0.075]	0.049	−0.002	−0.001
Equal loadings and thresholds	229.18 (126)	0.978	0.976	0.057 [0.045, 0.069]	0.050	0.002	−0.006

*Note*: The configural model specified the same factor structure across girls and boys without equality constraints. The equal‐thresholds model constrained item thresholds to equality across groups. The equal‐loadings‐and‐thresholds model constrained both factor loadings and item thresholds to equality across groups. Analyses used the WLSMV estimator for ordered categorical indicators; reported fit statistics are scaled estimates. Changes in fit are relative to the preceding model. Measurement invariance was considered supported when decreases in CFI did not exceed 0.010 and increases in RMSEA did not exceed 0.015 (Chen 2007).

Abbreviations: CFI = Comparative Fit Index; RMSEA = root mean square error of approximation; SRMR = standardized root mean square residual; TLI = Tucker–Lewis index.

## 4 Discussion

### 4.1 Overview of Findings

The present study provides initial evidence regarding the psychometric properties of the Persian MEWS‐12 among Iranian adolescents. Exploratory and confirmatory factor analyses favored a correlated two‐factor model over the unidimensional model, although the high correlation between the factors indicated substantial overlap between the two dimensions. The MEWS‐12 showed good internal consistency and was associated with depressive symptoms, anxiety symptoms, rumination, negative affect, mindfulness, and positive affect in generally expected directions, whereas its association with self‐reported attentional control was small and nonsignificant. Adolescents scoring at or above the selected anxiety or depressive symptom thresholds had higher MEWS‐12 scores than those scoring below these thresholds.

### 4.2 Factor Structure and Cultural Considerations

The factor analyses favored a correlated two‐factor representation of the Persian MEWS‐12 over the unidimensional model. However, the high correlations between the factors in both EFA and CFA indicate substantial overlap and suggest that the two factors should not be interpreted as clearly distinct dimensions. The two‐factor model was retained over the three‐factor model based on the parallel‐analysis findings, comparative model fit, and parsimony, whereas the bifactor model yielded an improper solution and could not be interpreted. Exploratory analyses of the original 15‐item pool did not yield a factor structure that was replicated in the independent CFA subsample; therefore, no alternative 15‐item structure was retained for interpretation.

The observed two‐factor representation did not fully reproduce the unidimensional structure reported in the original MEWS study (Mowlem et al. 2019) or the three‐factor representation previously examined for the MEWS‐12 (Morillas‐Romero et al. 2023). Such differences may reflect variation in samples, language, or analytic procedures, and the present data do not allow these possibilities to be distinguished.

The MEWS‐12 also showed good internal consistency at the total‐score level (Cronbach's *α* = 0.893; McDonald's *ω* = 0.917). These reliability estimates indicate consistency of the total score but do not, by themselves, resolve the dimensionality of the scale.

Cultural or linguistic contexts may influence responses to translated measures (Beaton et al. 2000; Van de Vijver and Leung 2021); however, no direct cross‐cultural comparison was conducted in the present study. Accordingly, the observed factor structure should not be interpreted as evidence of a culturally specific structure of excessive mind‐wandering.

### 4.3 Associations With External Measures and Symptom‐Defined Groups

The pattern of associations with external measures was generally consistent with expectations. Higher MEWS‐12 scores were associated with higher depressive and anxiety symptoms, rumination, and negative affect, and with lower mindfulness and positive affect. The associations with affective symptoms and mindfulness are broadly consistent with previous findings linking mind‐wandering with anxiety and depressive symptoms, affective experience, and mindfulness (Belardi et al. 2022; Fell et al. 2023; Webb et al. 2021; Welhaf et al. 2024).

In contrast, the association between MEWS‐12 scores and self‐reported attentional control was small and nonsignificant. Given the modest internal consistency of the ACS in the present sample, this finding should be interpreted cautiously.

Adolescents scoring at or above the selected anxiety or depressive symptom thresholds had higher MEWS‐12 scores than those scoring below the respective thresholds. Because group classification was based on questionnaire thresholds rather than diagnostic interviews, these findings should be interpreted as symptom‐based and not as evidence of diagnostic or screening validity.

### 4.4 Measurement Invariance Across Gender

Measurement invariance analyses provided preliminary support for comparable measurement properties of the MEWS‐12 across girls and boys at the tested levels. Changes in model fit after imposing equality constraints on thresholds and factor loadings remained within the prespecified criteria for invariance, although replication in independent samples is warranted.

### 4.5 Implications for Research and Practice

The present findings provide a basis for further research using the Persian MEWS‐12 among Iranian adolescents. The observed associations with emotional symptoms, rumination, affect, and mindfulness suggest that the measure may be informative in studies examining excessive mind‐wandering in relation to these constructs. Given the substantial overlap between the two factors identified in the present study, separate factor scores should be interpreted cautiously until the factor structure is replicated in independent samples. The present study does not establish diagnostic or screening utility; dedicated validation in clinical samples using appropriate reference standards would be required before any such application could be considered.

### 4.6 Limitations

Several limitations should be considered when interpreting the present findings. First, recruitment through schools and online platforms may limit the generalizability of the findings to other adolescent populations. In addition, reliance on self‐report measures and the cross‐sectional, single‐assessment design did not allow evaluation of test–retest reliability or longitudinal stability.

The psychometric evidence available for some of the Persian external measures was derived primarily from adult or older adolescent samples, and the ACS showed modest internal consistency in the present sample.

The GAD‐7 and PHQ‐9 score thresholds were used to form symptom‐based comparison groups rather than diagnostically defined groups. Because no diagnostic reference standard was used, the known‐groups findings should be interpreted as evidence of differentiation between symptom‐defined groups and not as evidence of diagnostic or screening validity.

Although expert review and pilot testing were conducted to evaluate the linguistic and cultural appropriateness of the Persian MEWS, formal quantitative content‐validity indices were not obtained. Future studies could further examine the factor structure, test–retest reliability, and content validity of the Persian MEWS‐12 and evaluate its performance in clinically characterized adolescent samples using appropriate reference standards.

## 5 Conclusions

The present study provides preliminary evidence regarding the psychometric properties of the Persian MEWS‐12 among Iranian adolescents. Exploratory and confirmatory analyses favored a correlated two‐factor representation over the unidimensional model, although the substantial correlation between the factors indicates considerable overlap between the two dimensions. The MEWS‐12 showed good internal consistency, and its associations with depressive and anxiety symptoms, rumination, negative affect, positive affect, and mindfulness were generally consistent with expectations. Adolescents scoring at or above the selected anxiety or depressive symptom thresholds also reported higher MEWS‐12 scores than those scoring below these thresholds, although these findings should not be interpreted as evidence of diagnostic or screening validity. Measurement invariance analyses provided preliminary support for comparable measurement properties across girls and boys. Overall, the Persian MEWS‐12 may be a useful measure for research on excessive mind‐wandering in Iranian adolescents, while further replication in independent, longitudinal, and clinically characterized samples is warranted.

## Author Contributions

**Mina Hasanpour**: conceptualization, validation. **Kousar Dadkhah Tehrani**: conceptualization, project administration. **Amir Sam Kianimoghadam**: supervision, writing – original draft. **Yalda Daneshi Hir**: writing – original draft, investigation. **Abolfazl Chekaneh Bajestani**: writing – review and editing, writing – original draft. **Shima Hasanpour**: software, formal analysis. **Maryam Maadi Esfahan**: writing – review and editing, supervision. All authors revised and approved the final version of the manuscript.

## Funding

This study was financially supported by the Research Department of the School of Medicine at Shahid Beheshti University of Medical Sciences (Grant Number 43011341).

## Ethics Statement

The study was conducted in accordance with the Declaration of Helsinki and approved by the Ethics Committee of Shahid Beheshti University of Medical Sciences (IR.SBMU.MSP.REC.1403.510). Written informed consent was obtained from parents or legal guardians, and assent was obtained from adolescent participants.

## Conflicts of Interest

The authors declare no conflicts of interest.

## Supporting information




**Supplementary Material**: brb371824‐sup‐0001‐SuppMat.docx

## Data Availability

The datasets used and analyzed during the current study are available from the corresponding author upon reasonable request.

## References

[brb371824-bib-0001] Abasi, I. , P. Mohammadkhani , A. Pourshahbaz , and B. Dolatshahi . 2017. “The Psychometric Properties of Attentional Control Scale and Its Relationship With Symptoms of Anxiety and Depression: A Study on Iranian Population.” Iranian Journal of Psychiatry 12, no. 2: 109–117. PMC5483236 28659983

[brb371824-bib-0002] Anthoine, E. , L. Moret , A. Regnault , V. Sébille , and J. B. Hardouin . 2014. “Sample Size Used to Validate a Scale: A Review of Publications on Newly‐Developed Patient Reported Outcomes Measures.” Health and Quality of Life Outcomes 12, no. 1: 2. 10.1186/s12955-014-0176-2. PMC4275948 25492701

[brb371824-bib-0003] Baer, R. A. , G. T. Smith , J. Hopkins , J. Krietemeyer , and L. Toney . 2006. “Using Self‐Report Assessment Methods to Explore Facets of Mindfulness.” Assessment 13, no. 1: 27–45. 10.1177/1073191105283504. 16443717

[brb371824-bib-0004] Beaton, D. E. , C. Bombardier , F. Guillemin , and M. B. Ferraz . 2000. “Guidelines for the Process of Cross‐Cultural Adaptation of Self‐Report Measures.” Spine 25, no. 24: 3186–3191. 10.1097/00007632-200012150-00014. 11124735

[brb371824-bib-0005] Belardi, A. , L. Chaieb , A. Rey‐Mermet , et al. 2022. “On the Relationship Between Mind Wandering and Mindfulness.” Scientific Reports 12, no. 1: 7755. 10.1038/s41598-022-11594-x. PMC9095883 35546599

[brb371824-bib-0006] Brislin, R. W. 1970. “Back‐Translation for Cross‐Cultural Research.” Journal of Cross‐Cultural Psychology 1, no. 3: 185–216. 10.1177/135910457000100301.

[brb371824-bib-0007] Carriere, J. S. , P. Seli , and D. Smilek . 2013. “Wandering in Both Mind and Body: Individual Differences in Mind Wandering and Inattention Predict Fidgeting.” Canadian Journal of Experimental Psychology / Revue canadienne de psychologie expérimentale 67, no. 1: 19–31. 10.1037/a0031438. 23458548

[brb371824-bib-0008] Chen, F. F. 2007. “Sensitivity of Goodness of Fit Indexes to Lack of Measurement Invariance.” Structural Equation Modeling: A Multidisciplinary Journal 14, no. 3: 464–504. 10.1080/10705510701301834.

[brb371824-bib-0009] Dekkers, T. J. , A. Flisar , A. Karami Motaghi , A. Karl , M. A. Frick , and B. E. Boyer . 2025. “Does Mind‐Wandering Explain ADHD‐Related Impairment in Adolescents?” Child Psychiatry & Human Development 56, no. 2: 346–357. 10.1007/s10578-023-01557-2. PMC11928403 37382726

[brb371824-bib-0010] Derryberry, D. , and M. A. Reed . 2002. “Anxiety‐Related Attentional Biases and Their Regulation by Attentional Control.” Journal of Abnormal Psychology 111, no. 2: 225–236. 10.1037/0021-843X.111.2.225. 12003445

[brb371824-bib-0011] Farrahi, H. , B. Gharraee , M. A. Oghabian , et al. 2021. “Psychometric Properties of the Persian Version of Patient Health Questionnaire‐9.” Iranian Journal of Psychiatry and Clinical Psychology 27, no. 2: 248–263. 10.32598/ijpcp.27.2.3375.1.

[brb371824-bib-0012] Fell, J. , L. Chaieb , and C. Hoppe . 2023. “Mind Wandering in Anxiety Disorders: A Status Report.” Neuroscience & Biobehavioral Reviews 155: 105432. 10.1016/j.neubiorev.2023.105432. 37898447

[brb371824-bib-0013] Heydarinasab, L. 2013. “An Investigation of the Validity and Reliability of Psychometric Characteristics of Five Facet Mindfulness Questionnaire in Iranian Non‐Clinical Samples.” International Journal of Behavioral Sciences 7, no. 3: 229–237.

[brb371824-bib-0014] Hu, L. T. , and P. M. Bentler . 1999. “Cutoff Criteria for Fit Indexes in Covariance Structure Analysis: Conventional Criteria Versus New Alternatives.” Structural Equation Modeling: A Multidisciplinary Journal 6, no. 1: 1–55. 10.1080/10705519909540118.

[brb371824-bib-0015] Khanjani, S. , A. A. Foroughi , M. Bazani , S. Rafiee , S. Tamannaeifar , and M. Habibi . 2022. “Psychometric Properties of Persian Version of Five Facets of Mindfulness Questionnaire.” Journal of Research in Medical Sciences 27, no. 1: 29. 10.4103/jrms.JRMS_10_20. PMC9081511 35548174

[brb371824-bib-0016] Kline, R. B. 2016. Principles and Practice of Structural Equation Modeling, 4th ed. Guilford Press.

[brb371824-bib-0017] Kroenke, K. , R. L. Spitzer , and J. B. Williams . 2001. “The PHQ‐9: Validity of a Brief Depression Severity Measure.” Journal of General Internal Medicine 16, no. 9: 606–613. 10.1046/j.1525-1497.2001.016009606.x. PMC1495268 11556941

[brb371824-bib-0018] Milfont, T. L. , and R. Fischer . 2010. “Testing Measurement Invariance Across Groups: Applications in Cross‐Cultural Research.” International Journal of Psychological Research 3, no. 1: 111–130. 10.21500/20112084.857.

[brb371824-bib-0019] Lanier, J. , E. Noyes , and J. Biederman . 2021. “Mind Wandering (Internal Distractibility) in ADHD: A Literature Review.” Journal of Attention Disorders 25, no. 6: 885–890. 10.1177/1087054719865781. 31364436

[brb371824-bib-0020] Liang, L. , and Y. H. Lee . 2019. “Factor Structure of the Ruminative Response Scale and Measurement Invariance Across Gender and Age Among Chinese Adolescents.” Advances in Applied Sociology 9, no. 6: 193–207. 10.4236/aasoci.2019.96016.

[brb371824-bib-0021] Luo, Y. , R. Zhu , E. Ju , and X. You . 2016. “Validation of the Chinese Version of the Mind‐Wandering Questionnaire (MWQ) and the Mediating Role of Self‐Esteem in the Relationship Between Mind‐Wandering and Life Satisfaction for Adolescents.” Personality and Individual Differences 92: 118–122. 10.1016/j.paid.2015.12.028.

[brb371824-bib-0022] MacCallum, R. C. , K. F. Widaman , S. Zhang , and S. Hong . 1999. “Sample Size in Factor Analysis.” Psychological Methods 4, no. 1: 84–99. 10.1037/1082-989X.4.1.84.

[brb371824-bib-0023] Mansouri, A. , A. Bakhshipour , M. M. Alilou , A. Farnam , and A. Fakhari . 2012. “The Comparison of Worry, Obsession and Rumination in Individual With Generalized Anxiety Disorder, Obsessive‐Compulsive Disorder, Major Depression Disorder and Normal Individual.” Psychological Studies 7, no. 4: 55–74. 10.22051/PSY.1970.1535.

[brb371824-bib-0024] Martinon, L. M. , J. Smallwood , C. Hamilton , and L. M. Riby . 2019. “Frogs' Legs Versus Roast Beef: How Culture Can Influence Mind‐Wandering Episodes Across the Lifespan.” Europe's Journal of Psychology 15, no. 2: 211. 10.5964/ejop.v15i2.1597. PMC7871756 33574952

[brb371824-bib-0025] Mohamadian, R. , H. Khazaie , S. M. Ahmadi , et al. 2022. “The Psychometric Properties of the Persian Versions of the Patient Health Questionnaires 9 and 2 as Screening Tools for Detecting Depression Among University Students.” International Journal of Preventive Medicine 13: 116. 10.4103/ijpvm.IJPVM_213_20. PMC9580551 36276890

[brb371824-bib-0026] Mokkink, L. B. , C. B. Terwee , D. L. Patrick , et al. 2010. “The COSMIN Checklist for Assessing the Methodological Quality of Studies on Measurement Properties of Health Status Measurement Instruments: An International Delphi Study.” Quality of Life Research 19, no. 4: 539–549. 10.1007/s11136-010-9606-8. PMC2852520 20169472

[brb371824-bib-0027] Mooneyham, B. W. , and J. W. Schooler . 2013. “The Costs and Benefits of Mind‐Wandering: A Review.” Canadian Journal of Experimental Psychology / Revue canadienne de psychologie expérimentale 67, no. 1: 11–18. 10.1037/a0031569. 23458547

[brb371824-bib-0028] Morillas‐Romero, A. , A. De la Torre‐Luque , F. D. Mowlem , and P. Asherson . 2023. “Spanish and Cross‐Cultural Validation of the Mind Excessively Wandering Scale.” Frontiers in Psychology 14: 1181294. 10.3389/fpsyg.2023.1181294. PMC10368185 37496793

[brb371824-bib-0029] Mowlem, F. D. , C. Skirrow , P. Reid , et al. 2019. “Validation of the Mind Excessively Wandering Scale and the Relationship of Mind Wandering to Impairment in Adult ADHD.” Journal of Attention Disorders 23, no. 6: 624–634. 10.1177/1087054716651927. PMC6429624 27255536

[brb371824-bib-0030] Mundfrom, D. J. , D. G. Shaw , and T. L. Ke . 2005. “Minimum Sample Size Recommendations for Conducting Factor Analyses.” International Journal of Testing 5, no. 2: 159–168. 10.1207/s15327574ijt0502_4.

[brb371824-bib-0031] Naeinian, M. , M. Shaeiri , M. Sharif , and M. Hadian . 2011. “To Study Reliability and Validity for a Brief Measure for Assessing Generalized Anxiety Disorder (GAD‐7).” Clinical Psychology and Personality 9, no. 1: 41–50.

[brb371824-bib-0032] Nolen‐Hoeksema, S. , and J. Morrow . 1991. “A Prospective Study of Depression and Posttraumatic Stress Symptoms After a Natural Disaster: The 1989 Loma Prieta Earthquake.” Journal of Personality and Social Psychology 61, no. 1: 115–121. 10.1037/0022-3514.61.1.115. 1890582

[brb371824-bib-0033] Ólafsson, R. P. , J. Smári , F. Guðmundsdóttir , G. Ólafsdóttir , H. L. Harðardóttir , and S. M. Einarsson . 2011. “Self Reported Attentional Control With the Attentional Control Scale: Factor Structure and Relationship With Symptoms of Anxiety and Depression.” Journal of Anxiety Disorders 25, no. 6: 777–782. 10.1016/j.janxdis.2011.03.013. 21531115

[brb371824-bib-0034] R Core Team . 2025. R: A Language and Environment for Statistical Computing. R Foundation for Statistical Computing. https://www.R‐project.org/.

[brb371824-bib-0035] Rashtbari, A. , M. Dadashi , M. Vakili , and H. Malekizadeh . 2019. “Valuation of the Prevalence of Generalized Anxiety Disorder and Its Association With Worry in Students of Zanjan University of Medical Sciences.” Journal of Medical Education Development 12, no. 36: 25–31.

[brb371824-bib-0036] Rosseel, Y. , T. D. Jorgensen , and L. De Wilde . 2026. “Lavaan: Latent Variable Analysis.” R Package Version 0.7‐2. CRAN. https://CRAN.R‐project.org/package=lavaan.

[brb371824-bib-0037] Sawyer, S. M. , P. S. Azzopardi , D. Wickremarathne , and G. C. Patton . 2018. “The Age of Adolescence.” Lancet Child & Adolescent Health 2, no. 3: 223–228. 10.1016/S2352-4642(18)30022-1. 30169257

[brb371824-bib-0038] Seli, P. , E. F. Risko , D. Smilek , and D. L. Schacter . 2016. “Mind‐Wandering With and Without Intention.” Trends in Cognitive Sciences 20, no. 8: 605–617. 10.1016/j.tics.2016.05.010. PMC5004739 27318437

[brb371824-bib-0039] Shin, K. M. , S. M. Cho , and K. H. Kim . 2015. “A Validation Study of the Korean‐Ruminative Response Scale in Korean Adolescents.” Psychiatry Investigation 12, no. 4: 508. 10.4306/pi.2015.12.4.508. PMC4620308 26508962

[brb371824-bib-0040] Shokri, O. , A. Goudarzi , and M. Sharifi . 2014. “Testing for Factorial Invariance of the Farsi Version of the Positive and Negative Affect Schedule Across Sexes.” Contemporary Psychology, Biannual Journal of the Iranian Psychological Association 9, no. 1: 83–94.

[brb371824-bib-0041] Smallwood, J. , and J. W. Schooler . 2015. “The Science of Mind Wandering: Empirically Navigating the Stream of Consciousness.” Annual Review of Psychology 66, no. 1: 487–518. 10.1146/annurev-psych-010814-015331. 25293689

[brb371824-bib-0042] Spitzer, R. L. , K. Kroenke , J. B. Williams , and B. Löwe . 2006. “A Brief Measure for Assessing Generalized Anxiety Disorder: the GAD‐7.” Archives of Internal Medicine 166, no. 10: 1092. 10.1001/archinte.166.10.1092. 16717171

[brb371824-bib-0043] Van de Vijver, F. J. , and K. Leung . 2021. Methods and Data Analysis for Cross‐Cultural Research. Cambridge University Press. 10.1017/9781107415188.

[brb371824-bib-0044] Vannucci, M. , C. Chiorri , A. Nocentini , and E. Menesini . 2020. “Distinguishing Spontaneous From Deliberate Mind Wandering in Adolescents: The Role of Attentional Control and Depressive Symptoms.” British Journal of Developmental Psychology 38, no. 3: 434–441. 10.1111/bjdp.12325. 32096263

[brb371824-bib-0045] Watson, D. , L. A. Clark , and A. Tellegen . 1988. “Development and Validation of Brief Measures of Positive and Negative Affect: The PANAS Scales.” Journal of Personality and Social Psychology 54, no. 6: 1063–1070. 10.1037/0022-3514.54.6.1063. 3397865

[brb371824-bib-0046] Webb, C. A. , E. S. Israel , E. Belleau , L. Appleman , E. E. Forbes , and D. A. Pizzagalli . 2021. “Mind‐Wandering in Adolescents Predicts Worse Affect and Is Linked to Aberrant Default Mode Network–Salience Network Connectivity.” Journal of the American Academy of Child & Adolescent Psychiatry 60, no. 3: 377–387. 10.1016/j.jaac.2020.03.010. PMC7736484 32553785

[brb371824-bib-0047] Webb, C. A. , A. O. Tierney , H. A. Brown , E. E. Forbes , D. A. Pizzagalli , and B. Ren . 2022. “Spontaneous Thought Characteristics Are Differentially Related to Heightened Negative Affect Versus Blunted Positive Affect in Adolescents: An Experience Sampling Study.” JCPP Advances 2, no. 4: e12110. 10.1002/jcv2.12110. PMC9937432 36817188

[brb371824-bib-0048] Welhaf, M. S. , J. Mata , S. M. Jaeggi , et al. 2024. “Mind‐Wandering in Daily Life in Depressed Individuals: An Experience Sampling Study.” Journal of Affective Disorders 366: 244–253. 10.1016/j.jad.2024.08.111. PMC11826933 39181165

