# Supplementary material for: Psychometric Properties of the Persian Version of the Mind Excessively Wandering Scale in Iranian Adolescents: A Validation Study
Source: Brain Behav. 2026 Oct 6;16(10):e71824. doi: 10.1002/brb3.71824 (PMC13642566; doi:10.1002/brb3.71824)
Supplement: Supplementary file 1 [file BRB3-16-e71824-s001.docx]

**Supplementary analyses of the 15-item MEWS**

To examine the factor structure of the originally administered 15-item MEWS, exploratory and confirmatory analyses were conducted in independent subsamples. In the EFA subsample (*n* = 254), sampling adequacy was high (KMO = .93), and Bartlett’s test of sphericity was significant, χ²(105) = 1980.72, *p* < .001. Parallel analysis based on polychoric correlations and 1,000 simulated datasets suggested retaining two factors. Specifically, the observed second factor eigenvalue (.47) exceeded the corresponding simulated eigenvalue (.36), whereas the observed third eigenvalue (.25) did not exceed its simulated counterpart (.29).

MINRES EFA with oblimin rotation showed that the two-factor solution fit better than the one-factor solution and had the lowest BIC. However, the factors were substantially correlated (*r* = .70). **A three-factor solution did not provide a clear advantage in fit or parsimony and was not retained for further evaluation.** Therefore, only the one- and two-factor solutions were evaluated in the independent CFA subsample (*n* = 252).

Using WLSMV estimation for ordinal indicators, neither the one-factor nor the EFA-derived two-factor model demonstrated acceptable robust fit. Although the two-factor model showed a small improvement over the one-factor model, its fit remained inadequate, χ²scaled(89) = 198.65, CFIrobust = .876, TLIrobust = .854, RMSEArobust = .099, 90% CI [.083, .116], SRMR = .063. The latent correlation between the two factors was high (*r* = .88). Item 14 showed a low standardized loading (.393), and Item 10 showed a comparatively weak loading (.489). Thus, the exploratory two-factor pattern of the 15-item version was not replicated in the independent CFA subsample.

**Table S1.** Exploratory factor analyses of the 15-item MEWS in the EFA subsample (n = 254)

| Model | χ² | df | TLI | RMSEA (90% CI) | RMSR | BIC |
| --- | --- | --- | --- | --- | --- | --- |
| One-factor | 290.48 | 90 | .875 | .094 [.082, .106] | .039 | −207.88 |
| Two-factor | 195.45 | 76 | .912 | .079 [.065, .093] | .030 | −225.39 |
| Three-factor | 163.57 | 63 | .910 | .079 [.065, .094] | .026 | −185.28 |

***Note****.* Extraction method = MINRES; two- and three-factor solutions were oblimin rotated. Parallel analysis based on polychoric correlations suggested two factors. The correlation between the two factors in the two-factor solution was .70.

**Table S2.** Confirmatory factor analyses of the 15-item MEWS in the independent CFA subsample (n = 252)

| model | χ²scaled | df | CFIrobust | TLIrobust | RMSEArobust (90% CI) | SRMR |
| --- | --- | --- | --- | --- | --- | --- |
| One-factor | 219.39 | 90 | .857 | .833 | .106 [.091, .122] | .066 |
| Two-factor | 198.65 | 89 | .876 | .854 | .099 [.083, .116] | .063 |

***Note****.* Estimator = WLSMV. The two-factor model was specified from the EFA pattern. The latent correlation between the two factors was .88.
